# Tectorigenin Attenuates Palmitate-Induced Endothelial Insulin Resistance via Targeting ROS-Associated Inflammation and IRS-1 Pathway

**DOI:** 10.1371/journal.pone.0066417

**Published:** 2013-06-19

**Authors:** Qi Wang, Xiao-Lan Cheng, Dong-Yan Zhang, Xue-Jiao Gao, Ling Zhou, Xiao-Ying Qin, Guo-Yong Xie, Kang Liu, Yong Qin, Bao-Lin Liu, Min-Jian Qin

**Affiliations:** 1 Department of Resource Science of Traditional Chinese Medicines, State Key Laboratory of Natural Medicines, China Pharmaceutical University, Nanjing, China; 2 Department of Pharmacy, Henan Provincial People's Hospital, Zhengzhou, China; 3 State Key Laboratory of Quality Research in Chinese Medicine, Institute of Chinese Medical Sciences, University of Macau, Taipa, Macao, China; 4 Department of Pharmacology of Chinese Materia Medica, China Pharmaceutical University, Nanjing, China; UAE University, Faculty of Medicine & Health Sciences, United Arab Emirates

## Abstract

Tectorigenin is a plant isoflavonoid originally isolated from the dried flower of *Pueraria thomsonii* Benth. Although its anti-inflammatory and anti-hyperglycosemia effects have been well documented, the effect of tectorigenin on endothelial dysfunction insulin resistance involved has not yet been reported. Herein, this study aims to investigate the action of tectorigenin on amelioration of insulin resistance in the endothelium. Palmitic acid (PA) was chosen as a stimulant to induce ROS production in endothelial cells and successfully established insulin resistance evidenced by the specific impairment of insulin PI3K signaling. Tectorigenin effectively inhibited the ability of PA to induce the production of reactive oxygen species and collapse of mitochondrial membrane potential. Moreover, tectorigenin presented strong inhibition effect on ROS-associated inflammation, as TNF-α and IL-6 production in endothelial cells was greatly reduced with suppression of IKKβ/NF-κB phosphorylation and JNK activation. Tectorigenin also can inhibit inflammation-stimulated IRS-1 serine phosphorylation and restore the impaired insulin PI3K signaling, leading to a decreased NO production. These results demonstrated its positive regulation of insulin action in the endothelium. Meanwhile, tectorigenin down-regulated endothelin-1 and vascular cell adhesion molecule-1 overexpression, and restored the loss of insulin-mediated vasodilation in rat aorta. These findings suggested that tectorigenin could inhibit ROS-associated inflammation and ameliorated endothelial dysfunction implicated in insulin resistance through regulating IRS-1 function. Tectorigenin might have potential to be applied for the management of cardiovascular diseases involved in diabetes and insulin resistance.

## Introduction

Diabetes mellitus is a devastating metabolic disorder and continues to be a major risk factor of morbidity and mortality throughout the world [1], [2]. Increasing evidence suggests that diabetes mellitus is closely linked with an increased risk of cardiovascular disease even in the presence of intensive glycemic control [3]. It was estimated that 75% of diabetic patients will die of cardiovascular disease (coronary heart disease, peripheral vascular disease and stroke) [3], [4]. Therefore, the management of diabetes has changed from glucocentric to organo protective and specially the vascular endothelium over the last two decades [5].

Insulin plays a key role in the regulation of glucose and lipid homeostasis in adipose tissue, skeletal muscle and liver. In addition to crucial metabolic actions, insulin exerts desirable effects on the maintenance of physiological endothelial function through its ability to stimulate NO release. This action was mainly mediated by a cascade of signaling that involves activation of the PI3K-Akt axis and the downstream activation of NO synthase (eNOS). Except NO-dependent vasodilatory actions, insulin also regulates proliferation, ET-1 and adhesion molecule expression in endothelial cells and this action is confirmed by increased insulin vasodilatory effects in humans under ET-1 receptor blockade [6]. Under insulin resistance conditions, PI3K-dependent signaling pathways were selectively impaired, leading to endothelial dysfunction, which is characterized by a decrease insulin-mediated NO production and increased secretion of ET-1 from the endothelium. Endothelial dysfunction and insulin resistance are frequently co-morbid states in diabetic patients, and are responsible for the increased risk of cardiovascular disease.

In oriental herbal medicine, the roots of *Pueraria thomsonii* Benth is well known as a Chinese traditional medicine for the management of diabetes and cardiovascular diseases, and the flowers of this plant (Fen-ge-hua in Chinese) have been traditionally used to counteract the overconsumption of alcohol [7]. A number of isoflavonoids have been isolated in this plant and their biological activities have been demonstrated [8]. The reported pharmacological effects of *P. thomsonii* Benth included preventive effect on hangovers [9], liver protective effects [10], antioxidant [11] and an estrogenic effect [12]. In addition, *P. thomsonii* Benth has been reported to lower serum lipid and blood glucose levels [13], [14], decrease body fat in humans [15]. Above-mentioned evidence suggests that *P. thomsonii* Benth might be efficacious against metabolic disorders, including diabetes mellitus.

Tectorigenin is a major isoflavonoid originally isolated from the dried flower of *P. thomsonii* Benth. This compound has been shown to possess hypoglycemic effect in streptozotocin-induced diabetic rats, and potently inhibited aldose reductase, suggesting tectorigenin is a promising compound for the prevention and/or treatment of diabetic complications [13], [16]. However, little is yet known about the effects of tectorigenin on the amelioration of insulin resistance involved in endothelial dysfunction. Since oxidative stress and inflammation play a pivotal role in the initiation of insulin resistance, this study investigated the effect of tectorigenin on NO production in endothelial cells with a focus on the link between reactive oxygen species (ROS)-associated inflammation and endothelial insulin resistance. The present findings demonstrate that tectorigenin attenuated insulin resistance (IR) associated with endothelial dysfunction through inhibiting ROS-related inflammation in endothelium cells, as well as facilitating insulin IRS-1/PI3K/Akt/eNOS signaling pathway.

## Materials and Methods

### Animals

Male Sprague-Dawley rats (200–250 g), supplied by the Laboratory Animal Center of Nanjing Qinglongshan (Certification number: SCXK 2009-0002). The care and treatment of these rats was maintained in accordance with the Provisions and General Recommendation of Chinese Experimental Animals Administration Legislation. Experimenters have License of Staff Engaged in Laboratory Animal (No. 2102044).

### Reagents

Tectorigenin (98.5% in purity) was isolated from the *Puerariae thomsonii* flos, following the previously reported methods of Wang et al. [17]. The identity of these compounds was confirmed by melting point, UV, IR, ^1^H and ^13^C NMR, and MS. The purity was determined to be higher than 98% by HPLC. In the present study, it was dissolved in DMSO before use. PA was obtained from Sinopharm Chemical Reagent Co., Ltd. (Shanghai, China) and dissolved in absolute ethanol at 200 mM as stock solution and then was further diluted with medium containing 10% FFA-free and low endotoxin BSA to obtain a concentration of 5 mM before use.

The following items were purchased from the cited commercial sources: RPMI Medium 1640 from Gibco (NY, USA); FBS from Sijiqing Biological Engineering Materials Co. Ltd. (Hangzhou, China); insulin from Wanbang Biochemical Pharmaceutical Company (Xuzhou, China); sodium salicylate from Tianjin Kemiou Chemical Agent Center (Tianjin, China); human TNF-α and IL-6 ELISA kits from R&D (USA), PI3K ELISA kits from Echelon Biosciences Inc. (SL city, USA). All primers from Sangon Biotec Co., Ltd. (Shanghai, China); TRNzol Plus, BU-Taq DNA polymerase, DNA Marker and BU-SuperScript RT kit from Biouniquer Technology Co., Ltd. (Nanjing, China). Anti-phospho-IRS-1 (Ser307), anti-IRS-1(R301), anti-phospho-Akt (T308), anti-Akt (A444), anti-phospho-IKKβ (Y199) and anti-IKKβ (F182) from Bioworld Technology (MN, USA); horseradish-peroxidase conjugated anti-rabbit and anti-mouse IgG antibodys, phospho-NF-κB p65 (Ser536) antibody, NF-κB p65 antibody, anti-SAPK/JNK, anti-phospho-SAPK/JNK (Thr183/Tyr185), phospho-eNOS (Ser 1177) antibody and eNOS (49G3) antibody from Cell Signaling Technology, Inc. (MA city, USA); PY99antibody from Santa Cruz biotechnology, inc.(CA, USA); glutathione reduced (GSH), Wortmannin and ECL Plus Western blotting detection reagent and Mitoquinone mesylate (MitoQ) were purchased from Beyotime Institute of Biotechnology (Shanghai, China), *4-hydroxypheny-L-glycine* from Sigma(St. Louis, MO, USA).

### Cell culture

Human umbilical vein endothelial cells (HUVECs) were purchased from the Type Culture Collection of the Chinese Academy of Sciences (Shanghai, China) and were examined morphologically and identified as endothelial cells by positive immunocytochemistry staining for Factor VIII. HUVECs were grown in RPMI Medium 1640 supplemented with 10% (*v*/*v*) FBS, streptomycin (100 U/ml) and penicillin (100 U/ml) under an atmosphere of 5% CO_2_ and 95% humidified air at 37°C. The medium was renewed every 2 days until the cells were grown to confluence.

### Measurement of intracellular ROS

Intracellular ROS generation was assessed using the ROS-specific fluorescent dye 2′, 7′-dichlorofluorescein, diacetate (DCFH-DA; Beyotime, Shanghai, China). When HUVECs were grown to conuence in 48-well plates, cells were washed three times with phosphate buffered solution (PBS) (8.0 g/L NaCl, 0.2 g/L KCl, 0.83 g/L Na_2_HPO_4_, 0.2 g/L KH_2_PO_4_, pH 7.2), and then were starved in serum-free medium for 12 h. After that, HUVECs were incubated with tectorigenin (0.1, 1, 10 μM), salicylate (5 mM), glutathione reduced (GSH, 1 mM), Mitoquinone mesylate (MitoQ, 0.1, 1 μM) for 30 min, then PA (100 μM) was added to the culture system for another 30 min. After washed with PBS and loaded with 10 μM DCFH-DA at 37°C for 30 min, cells were rinsed thrice with PBS and fixed in 2% paraformaldehyde (*v*/*v*) at 4°C for 5 min. Fixed cells were examined using an Olympus IX81 inverted microscope with attached charge-coupled device camera (Retiga Exi, Burnaby, BC, Canada) using appropriate filters with a peak excitation wavelength of 488 nm and a peak emission wavelength of 525 nm.

### NO production assay

NO production was assessed using the NO-specific fluorescent dye 3-Amino, 4-aminomethyl-2′,7′-difluorescein, diacetate (DAF-FM DA; Beyotime, Shanghai, China). When HUVECs cultured in 48-well plates reached conuence, cells were washed three times with PBS, then starved in serum-free medium for 12 h. Pretreated with tectorigenin (0.1, 1, 10 μM), salicylate (5 mM), glutathione reduced (GSH, 1 mM), Mitoquinone mesylate (MitoQ, 0.1, 1 μM) for 30 min, cells were stimulated without or with PA (100 μM) for 30 min and then washed thrice with PBS. For another experiment, cells were pretreated with tectorigenin (10 μM) for 30 min. After loading with 5 μM DAF-FM DA at 37°C for 30 min and kept in the dark, the HUVECs were rinsed three times with PBS. After that, cells were incubated with insulin (100 nM) at 37°C for 5 min, and fixed in 2% paraformaldehyde (vol/vol) at 4°C for another 5 min. Fixed cells were examined using an Olympus IX81 inverted microscope with attached charge-coupled device camera (Retiga Exi, Burnaby, BC, Canada) using appropriate filters with a peak excitation wavelength of 495 nm and a peak emission wavelength of 515 nm.

### Determination of the decreased mitochondrial membrane potential (Δψm)

Mitochondrial membrane potential production was assessed using the fluorescent dye 5,5′,6,6′-tetrachloro-1,1′,3,3′-tetraethylimidacarbocyanineiodide (JC-1; Beyotime, Shanghai, China). When HUVECs grown to conuence in 48-well plates, cells were washed three times with PBS, then starved in serum-free medium for 12 h. Pretreated with tectorigenin (0.1, 1, 10 μM), glutathione reduced (GSH, 1 mM) or Mitoquinone mesylate (MitoQ, 0.1 μM) for 30 min, cells were stimulated with PA (100 μM) for additional 30 min and then washed with PBS. After loading with 25 μg/ml JC-1 at 37°C for 30 min and kept in the dark, the HUVECs were rinsed three times with PBS and fixed in 2% paraformaldehyde (vol/vol) at 4°C for 5 min. In addition, a positive control which will induce potent collapse of mitochondrial membrane potential was designed. In this group, HUVECs were treated with 10 μM carbonylcyanide-m-chlorophenylhydrazone (CCCP) for 20 min without PA stimulation before loading with JC-1. Fixed cells were examined using an Olympus IX81 inverted microscope with attached charge-coupled device camera (Retiga Exi, Burnaby, BC, Canada) using appropriate filters with a peak excitation wavelength of 585 nm and a peak emission wavelength of 590 nm.

### Western blot analysis

HUVECs were serum-starved for 12 h and incubated with tectorigenin (0.1, 1, 10 μM), salicylate (5 mM) or GSH (1 mM) for 30 min, then stimulated with 100 μM PA for 30 min. After incubated in the absence or presence of insulin (100 nM) for 20 min, treated cells were washed with ice-cold PBS and lysed on ice in cell lysis buffer for western (product No. P0013, Beyotime, Shanghai, China) supplemented with 1 mM PMSF. Proteins were quantified using the Bicinchoninic Acid (BCA) Protein Assay kit (Biosky Biotechnology Corporation, Nanjing, China) in accordance with the manufacturer's instructions. Equal amounts of protein samples were separated by 10% SDS-PAGE and transferred to PVDF membranes (0.45 µm, Millipore Co., Ltd.) in a Mini Trans-Blot Electrophoretic Transfer Cell (Bio-Rad, USA). The membranes were blocked with 5% non-fat milk in TBST (5 mM Tris–HCl, pH 7.6, 136 mM NaCl, 0.05% Tween-20) for 2 h at room temperature, and probed with primary antibodies (1∶800 dilution in TBST) overnight at 4°C. Then incubated with the secondary antibody at 37°C for 1 h. HRP-labeled anti-rabbit or anti-mouse IgG was used as the secondary antibody (1∶2000 dilution in TBST). Signals were detected with an ECL western detection reagent and quantized by densitometry with Image-Pro Plus 6.0 (IPP 6.0) software.

### Determination of TNF-α and IL-6

HUVECs grown to confluence in 24-well plates were pretreated with tectorigenin (0.1, 1, 10 μM), salicylate (5 mM) or GSH (1 mM) for 30 min, then stimulated with PA (100 μM) for further 12 h in serum-free medium, and the medium was then collected on ice. The levels of TNF-α and IL-6 in the supernatant were assayed with commercial ELISA Kits.

### RNA extraction and RT-PCR analysis

For endothelin-1 (ET-1) and vascular cell adhesion molecule-1 (VCAM-1) gene determination, HUVECs were grown to confluence in six-well plates and then incubated with tectorigenin (0.1, 1, 10 μM), salicylate, wortmannin or PD98059 for 0.5 h, respectively, and then stimulated with PA for 2 h. After incubation, cells were treated with insulin for 20 min in the continued presence of the inhibitors or tectorigenin. Total RNA was extracted from the cells using TRNzol Plus and its concentration was calculated from spectrophotometric measurements at 260 nm. Total RNA (500 ng) from each RNA sample was reverse-transcribed into cDNA with the RT kit (Biouniquer Technology CO, LTD). Amplification of individual target cDNA was carried out with specific primers as followings: β-actin (161 bp), Forward primer: 5′-ACATCTGCTGGA AGGTGGAC-3′, Reverse primer: 5′-GGTACCACCATGTACCCAGG-3′; ET-1 (436 bp), forward primer: 5′-CGTTGTTCCGTATGGACTT G-3′; reverse primer: 5′-AGGCTATGGCTTCAGACA GG-3′; VCAM-1 (540 bp), forward primer: 5′-AGCCACATCAGTATCCAAGAGGAG-3′; reverse primer: 5′-CAATAACCAACTCTATGTTCCTTTTC-3′. Comparison to β-actin values was carried out for normalization. Relative expression levels in each sample were determined by comparison with the standard. The PCR products were electrophoresed in 1.5% agarose gel with 0.5 mg/mL ethidium bromide and visualized under UV light, then the signal intensity was detected by Bio-Rad image system and analyzed with computer program Quantity One (Bio-Rad).

### Preparation of aortic rings and assessment of endothelium-dependent relaxation

Rats were killed by cervical dislocation. The thoracic aorta was immediately removed and placed in 4°C Krebs-Henseleit (K-H) solution (NaCl 118.3, KCl 4.7, MgSO_4_ 1.2, KH_2_PO_4_ 1.2, CaCl_2_ 2.5, NaHCO_3_ 25, Calcium Disodium Edetate (EDTA) 0.026, glucose 5.0, mM, pH 7.4) and gassed with 95% O_2_ –5% CO_2_. The dissected aortae were cleaned of connective tissue and cut into rings (5 mm long). Take care to avoid abrading the intimal surface to maintain the integrity of the endothelial layer. For measurement of vascular responses, aortic rings were suspended in an organ bath containing 30 mL of K-H solution maintained at 37°C, pH 7.4, and continuously aerated with 95% O_2_ and 5% CO_2_. A resting tension of 2.0 g was applied to the aortic ring and changes in tension were measured with a force-displacement transducer connected to a polygraph. After an hour equilibration period, segments were exposed to 60 mM of KCl to assess the constriction function. The functionality of vascular endothelium was confirmed by the ability of relaxation after exposed to 10^−5^ M acetylcholine (Ach) to relax segments contracted with 10^−6^ M phenylephrine (the relaxation was over 80%). After confirmed the integrity of the endothelium, the aortic ring was pre-contracted with phenylephrine (10^−6^ M) and then insulin (10^−9^–10^−6^ M) was added cumulatively to evoke an endothelium-dependent relaxation. The relaxation induced by insulin was expressed as a percentage of the phenylephrine-induced contraction. After washout, the aortic ring was incubated with tectorigenin (0.1, 1, 10 μM) or salicylate (5 mM) for 30 min followed by the addition of PA (100 μM) for another 30 min. After the washout of tectorigenin and PA, the aortic ring was pre-contracted with phenylephrine (10^−6^ M). When the contraction was stable, increasing concentrations of insulin (10^−9^–10^−6^ M) were added to produce endothelium-dependent relaxation.

### Statistical analysis

All values were expressed as mean ± SD. Relevant data were performed with SPSS 15.0. For statistical analysis, we used the one-way ANOVA followed by Newman-Keuls test for multiple comparisons. Differences were considered significant at *p*<0.05.

## Results

### Tectorigenin suppresses ROS production in endothelial cells

**Figure 1:**
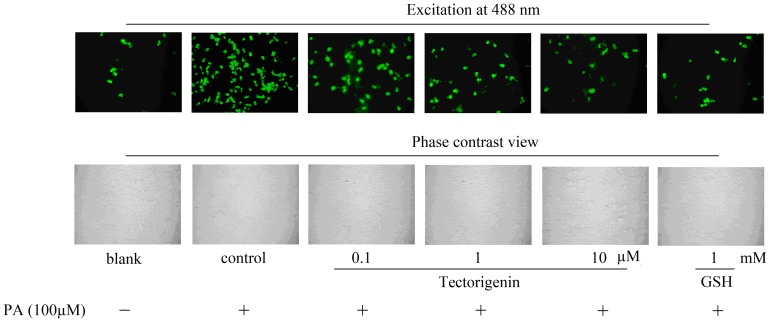
Tectorigenin decreased ROS production in PA-stimulated HUVECs. Cells were incubated with tectorigenin (0.1, 1, 10 μM), GSH or vehicle (medium containing 0.1%DMSO and 10% BSA) for 30 min, and then stimulated without o with PA (100 μM) for 30 min. Intracellular ROS fluorescence images were shown by using a fluorescence microscope, and the intensity of green fluorescence is used to assess ROS production. The GSH was taken as positive controls.

Because reactive oxygen species (ROS) is tightly associated with endothelial dysfunction, we first investigated the effect of tectorigenin on PA-induced ROS generation in HUVECs. After a 30 min treatment with PA (100 μM), intracellular oxidative stress was measured using a DCFH-DA fluorescent probe. Compared with the control, the fluorescence intensity in PA exposed cells was significantly higher, indicating ROS production was enhanced in endothelial cells. However, PA-stimulated ROS production was abolished by treatment with tectorigenin in a dose-dependent manner (0.1, 1, 10 μM). The inhibitory potency of tectorigenin at concentration of 10 μM was similar to that of 1 mM GSH (**Figure 1**), suggesting the potent antioxidant activity of tectorigenin in endothelial cells.

### Tectorigenin reversed PA-induced collapse of mitochondrial membrane potential (Δψm)

**Figure 2:**
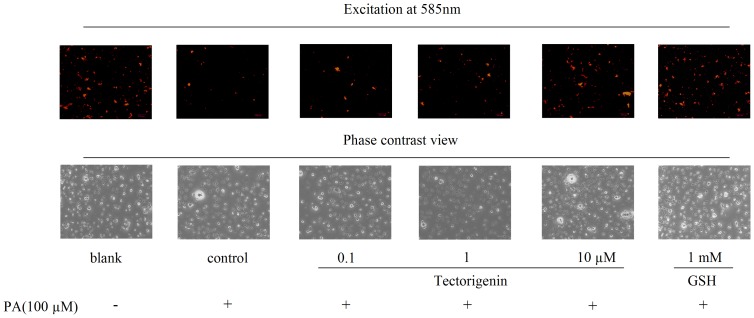
Tectorigenin reversed PA-induced collapse of mitochondrial membrane potential (Δψm) in HUVECs. Cells were pretreated with tectorigenin (0.1, 1, 10 μM) or GSH for 30 min, and then stimulated without or with PA (100 μM) for an additional 30 min. The blank was treated with an equal amount of the vehicle (medium containing 0.1% DMSO and 10% BSA). Fluorescence images were shown by using a fluorescence microscope, and the intensity of red fluorescence is used to assess Δψm.

The effect of tectorigenin on mitochondrial membrane potential of cells was determined by a JC-1 mitochondrial membrane potential assay kit. JC-1 is a fluorescent dye that is concentrated by respiring mitochondria. JC-1 exists as a monomer that fluoresces green and forms J-aggregates with red fluorescence at low and high Δψm, respectively. In this study, Δψm was measured in terms of the relative intensity of JC-1 after HUVECs were stimulated with PA and tectorigenin. **Figure 2** showed that PA stimulation decreased the mitochondrial membrane potential of HUVECs, implying the mitochondria damage. On the contrary, treatment of HUVECs with tectorigenin treament reversed this change evidenced by the restored Δψm. As a potent antioxidant, GSH was used as a positive control for the depolarized mitochondrial membrane potential and also showed a similar protective effect as tectorigenin.

### Tectorigenin inhibited IKKβ/NF-κB and JNK activation in PA-treated HUVECs

**Figure 3:**
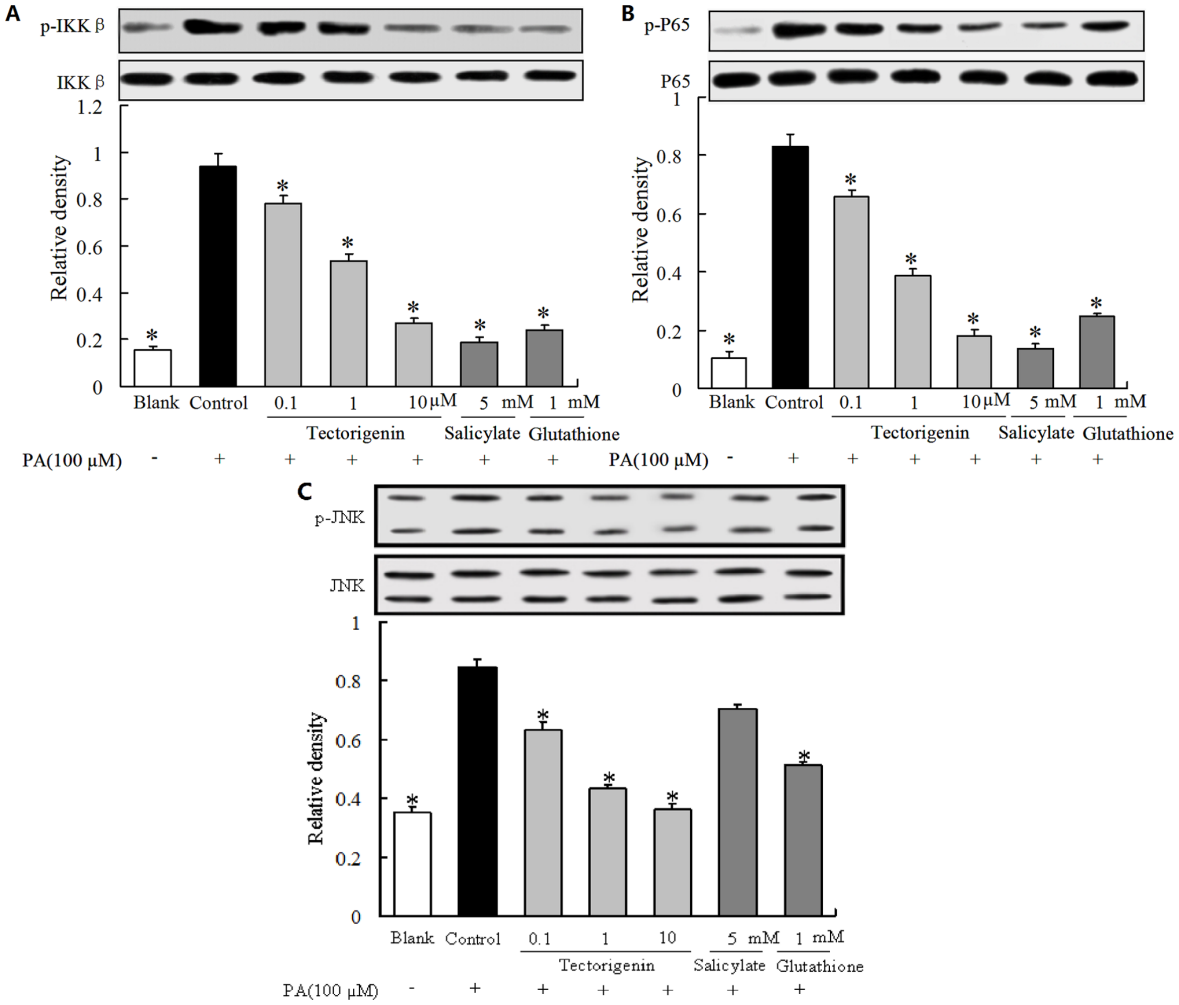
Tectorigenin inhibited IKKβ/NF-κB/JNK signaling in PA-treated HUVECs. (A–C): Cells were incubated with tectorigenin (0.1, 1, 10 μM), salicylate or GSH for 30 min, then stimulated without or with PA (100 μM) for 30 min. IKKβ (A), P65 (B) and JNK (C) phosphorylation were analyzed by Western blot. Salicylate and GSH were used as positive controls. The blank was treated with an equal amount of the vehicle (medium containing 0.1% DMSO and 10% BSA). The results were expressed as mean ± SD (*n* = 3). * p<0.05 vs control group.

IKKβ/NF-κB signaling is a key regulator of inflammation. As evidenced in **Figure 3A and B**, PA stimulation resulted in IKKβ and NF-κB phosphorylation activation. Treatment with tectorigenin attenuated enhanced IKKβ phosphorylation and effectively blocked NF-κB activation by inhibition of p65 phosphorylation at concentrations ranging from 0.1 to 10 μM. Salicylate and GSH also showed a similar inhibitory tendency on IKKβ and NF-κB activation evoked by PA stimulation. Meanwhile, we investigated the involvement of JNK in PA-induced inflammation in HUVECs. As shown in **Figure 3C**, PA stimulation increased JNK phosphorylation, and this action was reversed by tectorigenin. GSH treatment, but not salicylate, also effectively inhibited JNK phosphorylatin.

### Tectorigenin decreased TNF-α and IL-6 production in HUVECs

**Figure 4:**
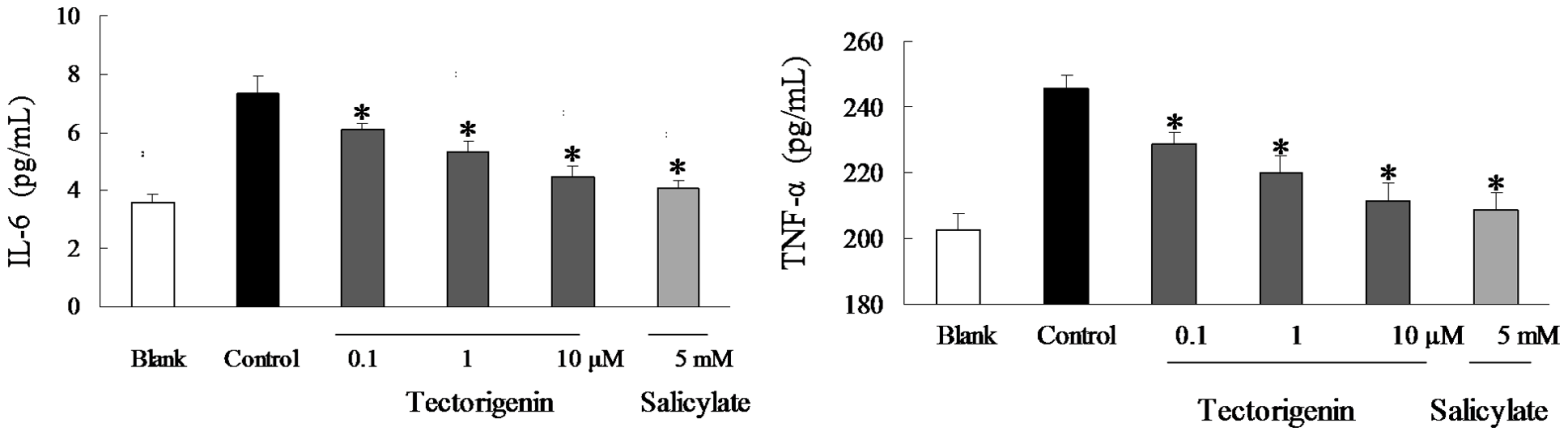
Tectorigenin inhibited IL-6 and TNF-α production in PA- stimulated HUVECs. HUVECs were pretreated with tectorigenin, salicylate for 30 min, and then incubated with PA (100 μM) for 12 h. The concentrations of TNF-α and IL-6 in the conditioned medium were measured with ELISA Kits. Salicylate was taken as positive control. The blank group was treated with an equal amount of the vehicle (medium containing 0.1% DMSO and 10% BSA). The results were expressed as the mean ± SD (*n* = 3). * p<0.05 vs control group.

IKKβ/NF-κB and JNK regulate the expression of a wide range of proinflammatory cytokines, including TNF-α and IL-6. As presented in **Figure 4**, TNF-α and IL-6 production were markedly increased when HUVECs were exposed to PA stimulation. Tectorigenin treatment effectively inhibited PA-augmented TNF-α and IL-6 production in a concentration dependent manner, demonstrating its anti-inflammatory action. Tectorigenin could suppress inflammatory response in the endothelium. As a positive control, salicylate also strongly inhibited TNF-α and IL-6 production evoked by PA stimulation (**Figure 4**).

### Tectorigenin modulated insulin receptor substrate-1(IRS-1) serine/tyrosine phosphorylation in the presence of PA

**Figure 5:**
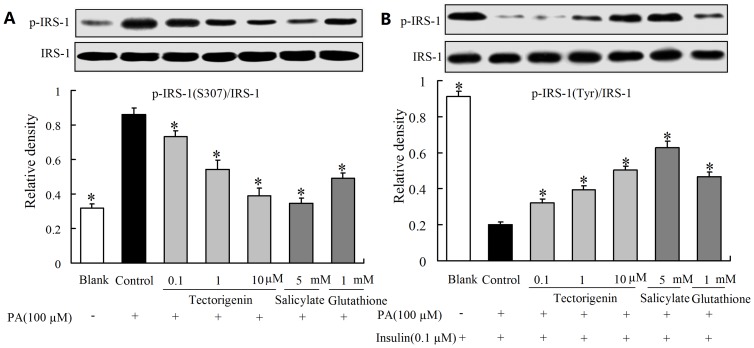
Tectorigenin modulated IRS-1 serine/tyrosine phosphorylation in PA-treated HUVECs. Cells were incubated with tectorigenin, salicylate or GSH for 30 min, and then stimulated without or with PA (100 μM) for 30 min. (A): Serine (S307) phosphorylation of IRS-1 was assayed by Western blot; (B): After stimulated with PA, HUVECs were then treated with insulin (100 nM) for 20 min. Tyrosine phosphorylation of IRS-1(detected by PY99 antibody) was then determined by Western blot. Salicylate and GSH were taken as positive controls. The blank was treated with an equal amount of the vehicle (medium containing 0.1% DMSO and 10% BSA). The results were expressed as mean ± SD of three independent experiments. * p<0.05 vs control group.

IRS-1 serine phosphorylation was a key molecular event linking inflammation to the impairment of insulin signaling [18]. PA stimulation induced serine phosphorylation of IRS-1(S307), and then attenuated IRS-1 tyrosine phosphorylation in response to insulin (detected by antibody of PY99). Exposure of endothelial cells to tectorigenin at concentrations ranging from 0.1 μM to 10 μM led to reduced PA-induced serine phosphorylation of IRS-1 and subsequently enhanced insulin-mediated IRS-1 tyrosine phosphorylation, indicating the beneficial regulation of IRS-1 function by tectorigenin (**Figure 5A and B**). Salicylate and GSH also demonstrated a similar modulation on IRS-1 serine and tyrosine phosphorylation as tectorigenin.

### Tectorigenin restored insulin-stimulated Akt and eNOS phosphorylation and NO prduction in the presence of PA

**Figure 6:**
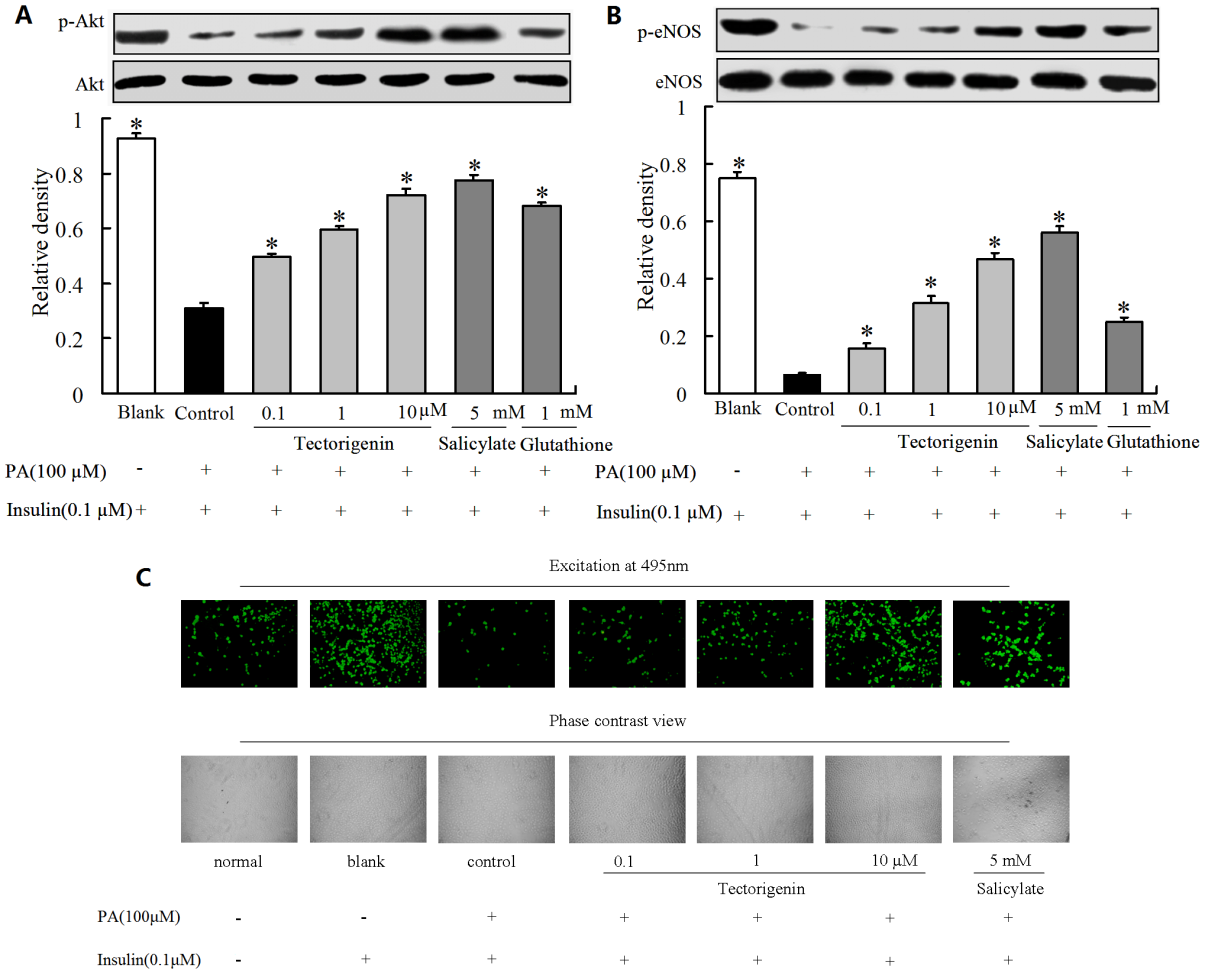
The effects of tectorigenin on insulin-mediated Akt and eNOS phosphorylation and NO production in HUVECs. (A–B): HUVECs were pretreated with tectorigenin, salicylate or GSH for 30 min, and then co-incubated with 100 μM PA for 30 min. After addition of insulin (100 nM) for 20 min, p-Akt (A) and p-eNOS (B) was determined by Western blot. The results were presented as mean ± SD of three independent experiments. * p<0.05 vs control group. (C) HUVECs were pretreated with tectorigenin or salicylate for 30 min, then incubated with 100 μM PA for another 30 min, followed by insulin (100 nM) stimulation for 5 min. Intracellular NO production was tested with fluorescence microscopy.

PA stimulation attenuated insulin-meidated IRS-1 tyrosine phosphorylaiton, leading to the impairment of downstream insulin signaling evidenced by reduced Akt and eNOS phosphorylation in response to insulin (**Figure 6A and B**). Tectorigenin pretreatment effectively mitigated the inhibitory effect of PA on insulin-mediated Akt/eNOS phosphorylation evidenced by restored Akt and eNOS phosphorylation (**Figure 6A and B**). Akt phosphorylation induces eNOS activation, which is directly responsible for the NO production in endothelium. PA impaired insulin signaling and blunted insulin action in promotion of NO production in endothelial cells. Thus, we further assessed levels of NO production in cells pretreated with PA, and observed that NO productions in response to insulin was reduced (**Figure 6C**). As expected, tectorigenin effectively protected cells against PA insult and restored the loss of insulin-mediated NO production.

### Tectorigenin inhibited ET-1 and VCAM-1 expression in HUVECs

**Figure 7:**
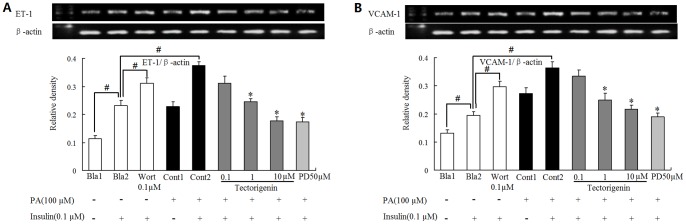
Tectorigenin reduced ET-1 and VCAM-1 expression in PA-stimulated endothelial cells. HUVECs were pretreated with tectorigenin, PD98059 or wortmannin (Wort) for 0.5 h and incubated with PA (100 μM) for another 2 h. Then cells were stimulated with or without insulin (0.1 μM) for 20 min. ET-1 (A) and VCAM-1 (B) mRNA expression was detected by RT-PCR. Salicylate was taken as a positive control. The results were expressed as the mean±SD of three independent experiments. ^#^ *p*<0.05 vs. blank 2 (Bla2); * *p*<.05 vs. control 2 (Cont2).

In addition to promoting NO production, insulin also stimulates ET-1 and adhesion molecule expression (VCAM-1) in endothelial cells through MAPK (ERK) pathways. The balance between PI3K-dependent and MAPK-dependent functions of insulin plays a key role in the maintenance of the endothelial homeostasis. In this study, insulin increased the basal expression of ET-1 and VCAM-1, and this action was enhanced by wortmannin treatment. When endothelial cells were exposed to PA, ET-1 and VCAM-1 expression in response to insulin was further increased. But tectorigenin effectively inhibited ET-1 and VCAM-1 overexpression in a concentration-dependent manner. PD98059, a specific inhibitor of ERK, also downregulated ET-1 and VCAM-1 expression as tectorigenin did, indicating the involvement of ERK activation. The results were shown in **Figure 7**A and **B**.

### Tectorigenin restored insulin-mediated vessel relaxation in rat aorta

**Figure 8:**
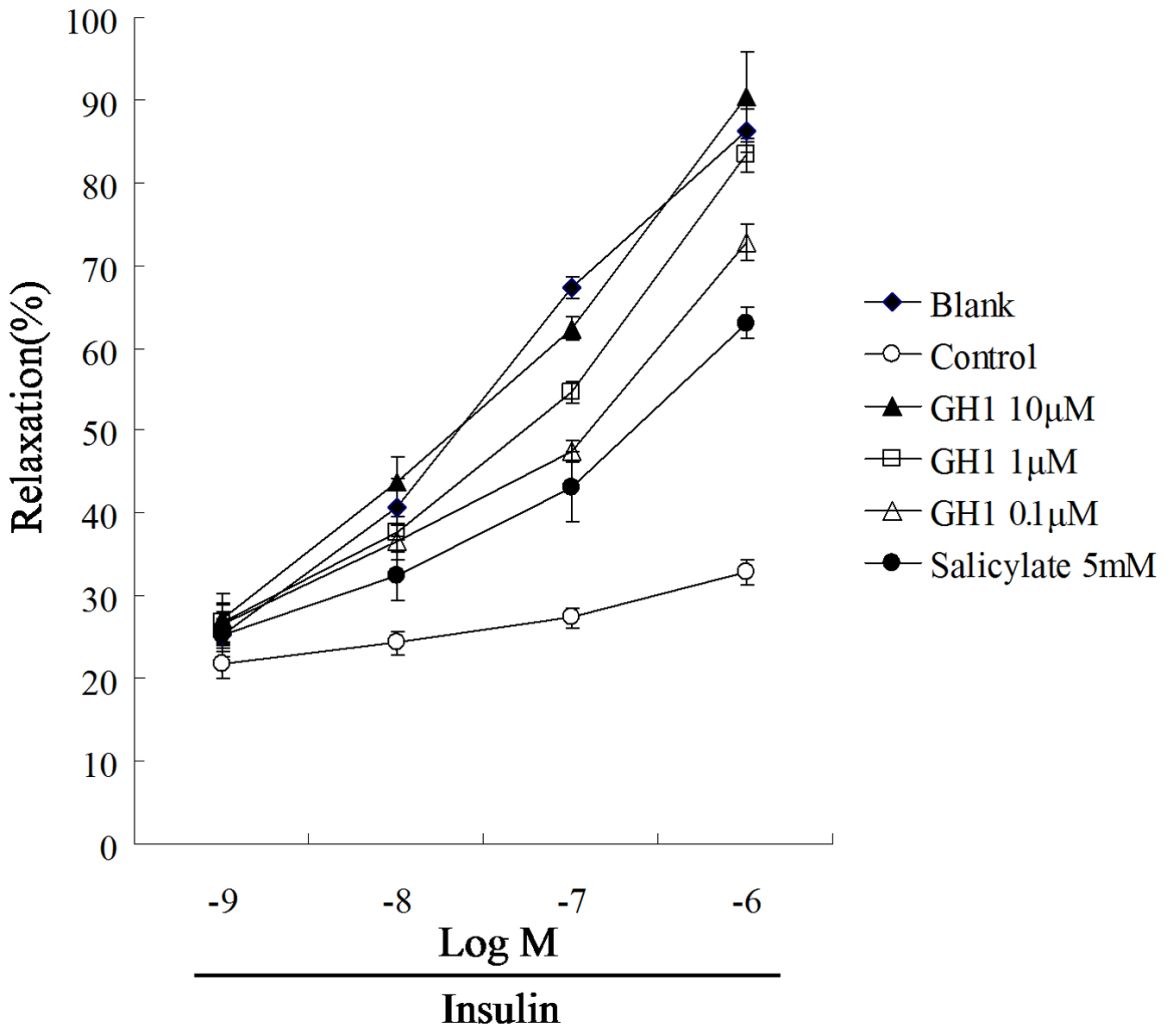
Tectorigenin restored PA-induced loss of endothelium-dependent vessel relaxation in the rat aorta. The aortic ring was pretreated with tectorigenin or salicylate for 30 min, then stimulated with PA (100 μM) for 30 min. After washing out of tectorigenin and PA, the aortic ring was pre-contracted with phenylephrine (10^−6^ M), and then the relaxation was induced by insulin treatment. Salicylate was used as a positive control. The blank was treated with an equal amount of the vehicle (medium containing 0.1% DMSO and 10% BSA). The results were expressed as the mean±SD (*n* = 4).

Endothelial dysfunction is characterized by the loss of endothelium-dependent vessel relaxation. Data presented in **Figure 8** showed that insulin induced vasodilation at concentrations ranging from 10^−9^ to 10^−6^ M. Whereas the stimulation of rat aorta with PA impaired insulin action, resulting significant decrease of relaxation. The percentages of relaxation induced by 0.1 μM and 1 μM of insulin were reduced from 67.28% to 15.35% (0.1 μM of insulin) and from 86.26% to 20.74% (1 μM of insulin), respectively. Tectorigenin treatment significantly resotored insulin-meidated vessel relaxation evidenced by largest relaxation (1 μM of insulin) were restored to 72.75%, 83.34% and 90.44% by tectorigenin at concentrations of 0.1, 1 and 10 μM. The result demonstrated that tectorigenin protected endothelium-dependent relaxation against inflammatory insult.

## Discussion

Oxidative stress, which may be precipitated by hyperglycemia and vascular inflammation, plays a pivotal role in the development of diabetes complications, both microvascular and cardiovascular. Hyperglycemic conditions of cells cause ROS overproduction and the deregulation of ROS signaling pathways, leading to impaired endothelial function and consequently to vascular dysfunction. Consequently, reducing oxidative stress by lowering ROS production is crucial in the management of diabetes complications. It has been established that excessive circulating free fatty acids (FFAs) stimulates ROS formation in endothelial cells through PKC activation [19]. FFAs also successfully established insulin resistance evidenced by the specific impairment of insulin PI3K signaling [20]. Tectorigenin presented effective suppression on PA-induced ROS overproduction and facilitated insulin action on the endothelium, well demonstrating its antioxidant potency and chemoprotection against FFA insult. There are several pathways involved in ROS production under the conditions of insulin resistance, including NADPH oxidase, xanthine oxidase, and mitochondria-mediated pathways. Mitochondrion is the main site of ROS production in the cell, and is sensitive to ROS with marked collapse in Δψm [21], [22]. It is reported that PA-induced ROS production is mainly derived from mitochondrial dysfunction [20]. In this regard, we investigated the effect of tectorigenin on mitochondrial integrity and functional loss in PA-treated HUVECs. As a result, marked collapse of Δψm in PA treated cells was observed due to increased ROS levels. Tectorigenin showed significant inhibition on ROS production, and reduced the collapse of mitochondrial Δψm in PA-treated HUVECs, suggesting its chemoprotection effect on mitochondrial function in the endothelium.

Oxidative stress is tightly concerned with inflammation, and ROS evoke inflammatory response *via* IKK/NF-κB and JNK/SAPK signaling pathways [23]. The proinflammatory pathway has been evoked in a variety of insulin resistant states. Tectorigenin was reported to inhibit the production of inflammatory mediators [24]. Thus, we wondered whether its anti-inflammatory activity contributed to attenuation of insulin resistance in the endothelium. Elevated FFAs level is an important pathological factor for the pathogenesis of both inflammation and insulin resistance in endothelium [25], so PA was used as a stimulus to induce inflammatory response in endothelial cells. IKKβ, a serine kinase, can modulate proinflammatory cytokine expression or production by activating NF-κB. In this study, PA induced IKKβ and NF-κB p65 phosphorylation, resulting in increased TNF-α and IL-6 production in endothelial cells (**Figure 1**). Treatment with tectorigenin suppressed PA-stimulated IKKβ/NF-κB activation, and decreased production of TNF-α and IL-6. These actions were similar to salicylate, an IKKβ inhibitor. Tectorigenin inhibited inflammation in an IKKβ/NF-κB-dependent manner. The result is also consistent with the published reports which showed that tectorigenin inhibited inflammation in macrophages by blocking NF-κB pathway [24]. The inhibition of tectorigenin on TNF-α and IL-6 production was, at least in part, responsible for its amelioration of endothelial dysfunction.

It has been well established that inflammation is a key feature of insulin resistance. And serine phosphorylation of insulin receptor substrate-1 (IRS-1) is a key event linking inflammation and insulin resistance. Inflammatory molecules such as TNF-α, IL-6 and IKKβ can affect IRS-1 function and impaired insulin signaling [26]. ROS have been shown to active JNK, which in turn phosphorylates IRS-1 at serine residues and disturbs tyrosine phosphorylation of IRS-1 in response to insulin [27]. To determine the effect of tectorigenin on restoring insulin signal transduction in vascular endothelium, we evaluated tyrosine phosphorylation of IRS-1. PA was observed to induce IRS-1 serine phosphorylation with down-regulated expression of IRS-1 tyrosine phosphorylation in response to insulin. However, these alterations were effectively reversed by tectorigenin, well demonstrating its beneficial modification of serine/tyrosine phosphorylation of IRS-1. In view of the involvement of inflammation and oxidative stress, the suppression of ROS production and IKKβ/NF-κB dependent inflammatory response in endothelial cells by tectorigenin should be responsible for its positive regulation of IRS-1 function.

Insulin resistance is a characteristic feature of the impairment of PI3K signaling. Binding of insulin to its receptor on endothelial cells leads to phosphorylation of IRS-1, which subsequent binds and activates PI3K.The activated PI3K phosphorylates and activates downstream Akt and eNOS, leading to NO production. PA inhibited insulin-mediated IRS-1 tyrosine phosphorylation, and subsequently impaired insulin signaling along the PI3K/Akt/eNOS pathway. As tectorigenin attenuated PA-induced IRS-1 serine phosphorylation and enhanced insulin mediated IRS-1 tyrosine phosphorylation, we reasoned that the phosphorylating modification of IRS-1 should contribute to the regulation of insulin PI3K signaling. As expected, tectorigenin restored insulin-mediated Akt and eNOS phosphorylation impaired by PA (**Figure 3A and B**). The improvement of Akt/eNOS phosphorylation by tectorigenin might be relative with its positive modulation of IRS-1 phosphorylation against PA insult. NO is important for modulating endothelial function. Deficiency of endothelial-derived NO is the primary defect that links insulin resistance and endothelial dysfunction. Since eNOS is an important producer of NO, the beneficial modulation of insulin PI3K/Akt/eNOS signaling by tectorigenin would contribute to the resultant improvement of insulin-stimulated NO production in endothelial cells. As the results shown, tectorigenin effectively protected cells against PA insult and restored the loss of insulin mediated NO production. Because loss of insulin-mediated vessel relaxation is a main characteristic of endothelial dysfunction under insulin resistant state, we studied the effect of tectorigenin on insulin-mediated vasodilation in isolated rat aortic rings. PA stimulation induced the loss of insulin-mediated vasodilation, but tectorigenin treatment was found to effectively reverse the effect of PA. The beneficial regulation of insulin PI3K signaling and increased NO production in the endothelium appears to be the key mechanisms of action by which tectorigenin exerts its benefit effects on restoration of insulin-mediated vasodilation.

**Figure 9:**
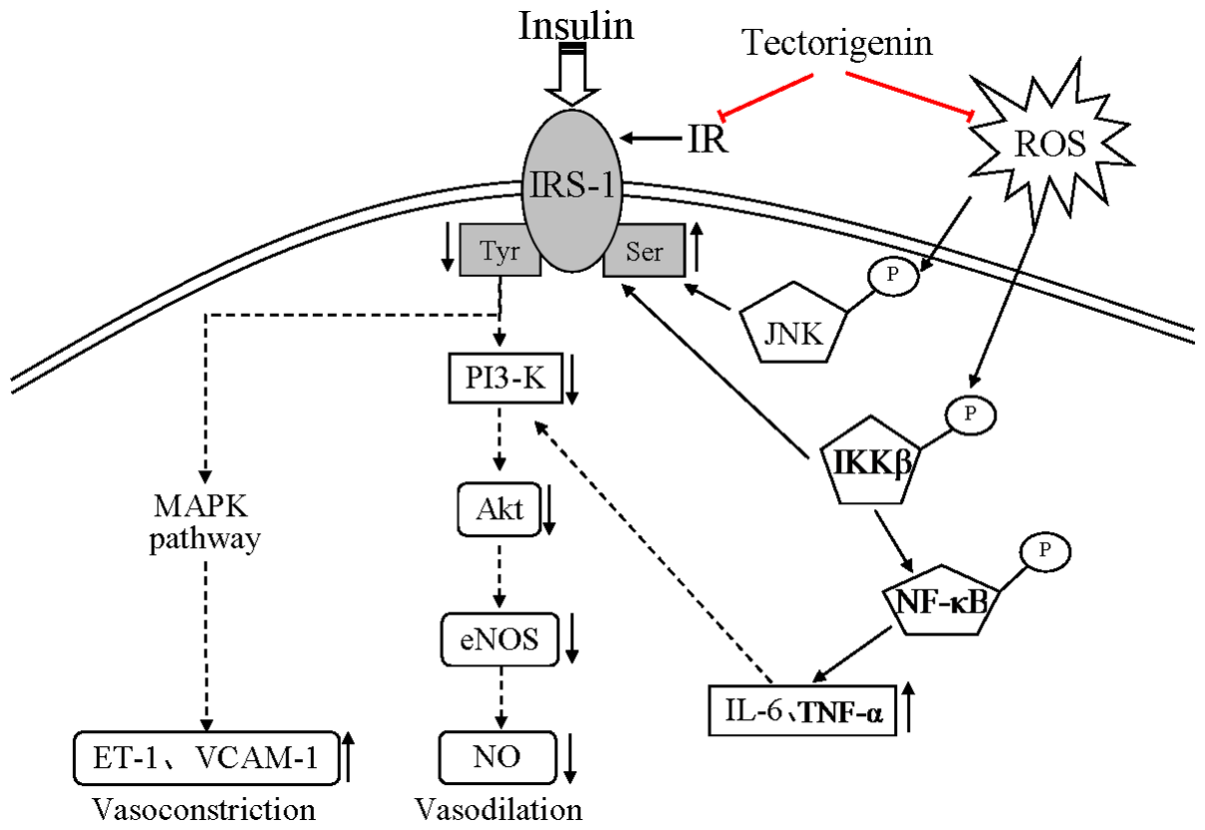
Schematic representation of the effects of tectorigenin to block hyperglycaemia-induced vascular endothelial dysfunction.

It has been proposed in various studies that insulin stimulates the release of both ET-1 and NO, and the hemodynamic effects of insulin are a balance between the vasodilator (NO) and the vasoconstrictor (ET-1) effects [28], [29]. Pathway-specific impairment in PI3K signaling in endothelium may decrease NO production and increase ET-1 expression. Insulin stimulates ET-1 and VCAM-1 in endothelial cells through MAPK (ERK) pathways. In this work, we treated cells with wortmannin, a PI3K inhibitor, and observed that insulin-stimulated ET-1 and VCAM-1 expression were upregualted. But PD98059, a specific inhibitor of ERK, down-regulated ET-1 and VCAM-1 expression. These results further confirmed that inhibition of PI3K enhanced the mitogenic action of insulin, and MAPK activation was involved in the expression of ET-1 and VCAM-1. PA stimulation has been shown to impair PI3K signaling, and this action would enhance MAPK-dependent actions of insulin. As a result, insulin-stimulated ET-1 and VCAM-1 expressions were greatly enhanced when cells were treated with PA. Tectorigenin treatment not only restored insulin-mediated NO production in a PI3Kdependent manner, but also inhibited ET-1 and VCAM-1 production. Therefore, tectorigenin played a definite positive effect on restoration of the balance between PI3K-dependent and MAPK dependent functions of insulin. A schematic diagram showing the proposed mechanisms by which tectorigenin inhibited endothelial dysfunction resulting from PA is shown in **Figure 9**.

The present study aimed to investigate the actions of tectorigenin on PA-induced endothelial dysfunction, and provide insight into the possible mechanisms. Tectorigenin inhibited ROS-associated inflammation and restored insulin signaling transduction along the IRS-1/PI3K /Akt/eNOS pathways. Meanwhile, tectorigenin suppressed enhanced mitogenic action of insulin by inhibition of ET-1 and VCAM-1 expression, well demonstrating its beneficial modulation of insulin actions in endothelium. The resulting increase in insulin-mediated NO should be responsible for actions of tectorigenin in amelioration of endothelial dysfunction. Taken together, the action of tectorigenin on attenuating endothelial dysfunction implicated in insulin resistance provides us new insights to its possible application for the prevention or treatment of cardiovascular disorders involved in insulin resistance and diabetes.
